# Supplementary material for: SARS-CoV-2 genetic diversity in Venezuela: Predominance of D614G variants and analysis of one outbreak
Source: PLoS One. 2021 Feb 19;16(2):e0247196. doi: 10.1371/journal.pone.0247196 (PMC7895374; doi:10.1371/journal.pone.0247196)
Supplement: Attachment — Submitted filename: Response to reviewers.docx [file pone.0247196.s002.docx]

PONE-D-20-31900R1
SARS-CoV-2 genetic diversity in Venezuela: predominance of D614G variants and analysis of one outbreak
There are still two points that need to be revised:

increase the number to 1000 bootstrap iterations for better analysis

Bootstrap iterations were increased to 1000. New Figure is included.

and all Figures need improvement in resolution and colors, in particular Figure 7.

Figures are now included with better resolution and colors were slightly modified in Figure 7, for better appreciation.

The Figure 6 needs to be done with two scales in order to identify the lowest values.

The scale of Figure 6 was changed to a logarithmic one for better evaluation of lowest values.
